# Hamp Type-1 Promotes Antimicrobial Defense via Direct Microbial Killing and Regulating Iron Metabolism in Grass Carp (*Ctenopharyngodon idella*)

**DOI:** 10.3390/biom10060825

**Published:** 2020-05-28

**Authors:** Yazhen Hu, Tomofumi Kurobe, Xiaoling Liu, Yong-An Zhang, Jianguo Su, Gailing Yuan

**Affiliations:** 1 Department of Aquatic Animal Medicine, College of Fisheries, Huazhong Agricultural University, Wuhan 430070, China; yazhenhu@webmail.hzau.edu.cn (Y.H.); liuxl@mail.hzau.edu.cn (X.L.); yonganzhang@mail.hzau.edu.cn (Y.-A.Z.); sujianguo@mail.hzau.edu.cn (J.S.); 2 Hubei Engineering Technology Research Center for Aquatic Animal Disease Control and Prevention, Wuhan 430070, China; 3 Department of Anatomy, Physiology and Cell Biology, School of Veterinary Medicine, University of California, Davis, CA 95616, USA; tkurobe@ucdavis.edu

**Keywords:** antibacterial, *Ctenopharyngodon idella*, hepcidin, host defense, iron homeostasis

## Abstract

Hepcidin is an antimicrobial peptide and regulator of iron homeostasis which has two isoforms in most fishes and some mammals. Previous studies have reported that the two hepcidin isoforms have different roles. Hamp type-1 plays a regulatory role in iron metabolism and hamp type-2 mostly performs an antimicrobial role. In this study, we found that *Ctenopharyngodon idella* (*C. idella*) have only one hepcidin isoform (hamp type-1), which showed both broad-spectrum antibacterial and iron regulatory functions. *C. idella* hepcidin mature peptide (hepcidin-25) and truncated peptide (hepcidin-20) exhibited bactericidal activities against both Gram-positive and Gram-negative bacteria in a dose-dependent manner in part through membrane rupture and binding to bacterial genomic DNA. The data from challenge tests demonstrated that the administration of hepcidin-25 significantly reduced mortality rates of *C. idella* by *A. hydrophila* infection, probably due to direct bactericidal activities of the peptide and a reduction of iron content in the fish serum. In addition, a comparison between hepcidin-20 and -25 suggests that the N terminal 5 amino acids play a critical role in reducing iron content in fish serum. Our findings revealed an important role of hamp type-1 in maintaining iron homeostasis and fighting against bacterial infections, suggesting the hepcidin has implications for the prevention and control of bacterial infection in aquaculture.

## 1. Introduction

Hepcidin is a cysteine-rich iron regulating peptide with a predicted complex disulfide pattern [1,2]. Hepcidin was originally identified in human blood as an antimicrobial peptide that was mainly expressed by the liver and released into serum [1,3]. In mammals, hepcidin exhibits strong bactericidal activity against various Gram-negative and Gram-positive bacteria [4,5]. As the research advanced, hepcidin was found to be a multifunctional molecule that plays an important role in maintaining iron homeostasis as well [6].

In mammals, hepcidin plays a critical role in host defense system against bacterial infections by inducing reactive hypoferremia during early phases of infection [7,8]. Physiologically, iron is an essential element involved in almost all living organisms, including bacteria, mammals, and plants [9]. During infections, pathogenic bacteria extract iron from host transferrin or lactoferrin via its iron-carrying proteins (transferrin and lactoferrin), which have a higher iron affinity than that of the other two host proteins [10]. However, hepcidin triggers iron efflux protein Ferroportin (Fpn) polyubiquitination and induces its internalization. These reactions result in the blocking of cellular iron efflux and reduce the available iron content of extracellular pathogens [6,11,12,13]. Thus, iron removal regulated by hepcidin is an important strategy for controlling bacterial growth and survival.

Hepcidin gene is highly conserved among different species in mammals, reptiles, and fishes and its copy number per genome can vary among different organisms. For example, humans and most mammals have only one hepcidin gene, which has both iron regulation and antibacterial activities. In contrast, most fish species have two or even more homologous hepcidin genes [14,15]. Zebrafish (*Danio rerio*) and rainbow trout (*Oncorhynchus mykiss*) have two homologous hepcidin genes [16,17], and yellow croaker (*Larimichthys crocea*) has more [18]. In general, fish hepcidin genes can be classified into two types: hamp type-1 and hamp type-2 [15,19]. Among the two types of hepcidin genes, studies have shown that the antibacterial function of fish hepcidin is mainly concentrated in type-2 whereas type-1 is responsible for iron regulation [16,20,21,22,23,24]. Fish hamp type-1 regulates iron metabolism, resulting in iron redistribution, decreasing serum iron levels, and increasing iron accumulation in hepatopancreas [25,26,27,28]. Hamp type-2 seems not to be related to iron metabolism. This further confirmed the differential roles of hamp type-1 and hamp type-2 as iron metabolism regulator and antimicrobial peptide, respectively [20,29].

The screening of the grass carp genome demonstrated that there is only hepcidin gene, hamp type-1, which led us to generate a hypothesis that the gene has both iron regulation and antibacterial activities similar to the one in humans and other mammals. To test the hypothesis, we assessed antibacterial and iron regulation activities of the grass carp hamp type-1 mature peptide (hepcidin-25) using chemically synthesized peptides. In addition, we evaluated the therapeutic and preventive effects of hepcidin against bacterial infection via in vitro and in vivo tests. Furthermore, truncated peptide lacking five amino acid residues in the N-terminal region (hepcidin-20) was chemically synthesized to elucidate its function in biological activities of hepcidin.

## 2. Materials and Methods

### 2.1. Ethical Statement

The use of experimental fish was approved by the Animal Ethics Committee of Huazhong Agricultural University. The approval number is HZAUFI-2019-014 (Approval date: 15 March 2019). The tissue material from fish was used for the present study did not involve endangered or protected species. All experimental animals were anesthetized with 3-Aminobenzoic acid ethyl ester methanesulfonate (MS-222), and every effort was made to minimize suffering.

### 2.2. Protein Modelling and Synthesis of Hepcidin Peptides

A *C. idella* hepcidin mature peptide (QSHLSLCRYCCNCCRNKGCGYCCKF) model was generated using the online software (https://swissmodel.expasy.org) based on the published protein sequence of hepcidin, while further modelling of hepcidin was based on published atomic structures of hepcidin. We synthesized the hepcidin peptides with (hepcidn-25) or without (hepcidin-20) the N-terminal sequences based on the structural characteristics predicted by protein modelling. Hepcidin-20 is lacking glutamine, serine, histidine, leucine, serine, and leucine. Using the air oxidation method and DMS methods, both peptides were synthesized by GenScript (Nanjing, China) and split into 1 mg every tube. All of the HPLC-purified peptides exhibited a purity of 95% (Supplementary Figure S1A,B).

### 2.3. Effects of Immunostimulants on Hepcidin mRNA Levels

The L8824 cells were exposed to various types of immunostimulant to evaluate gene expression of the hepcidin gene. The stimulants included heat-inactivated *A. hydrophila* (*hi A. h*), lipopolysaccharides (LPS), peptidoglycan (PGN), polyinosinic: polycytidylic acid (poly (I:C)). *hi A. h* was prepared by heating the intact bacteria at 100 °C for 45 min. The other stimulants were purchased from Sigma-Aldrich (Sigma, Saint Louis, MO, USA). L8824 cells were seeded in 12-well plates for 24 h adherence. Subsequently, cells were stimulated with 2 μg/mL LPS, PGN, poly (I:C), *A. hydrophila* DNA, or *hi A. h* for 3, 6, 12, and 24 h and the expression of hepcidin was measured by qRT-PCR.

Total RNA was isolated from cells, followed by cDNA synthesis. qRT-PCR was performed according to the manufacturer’s instructions using a Roche LightCycler^®^ 480 system and the 2 × T5 Fast qPCR Mix (SYBR Green I) (Beijing TsingKe Biotech Co., Ltd., Beijing, China). The primers used in this study were listed in Table 1. Data were analyzed using 2^−ΔΔCT^ method, and the housekeeping gene EF1α served as an internal control [30,31].

### 2.4. Plasmid Constructions and Transfections

Grass carp hepatopancreas (*L8824*) cell line, Fathead minnow (FHM) and Human embryonic kidney 293T (HEK293T) cells were obtained from the China Center for Type Culture Collection. L8824 and FHM cells were grown in M199 (Gibco, Grand Island, NY, USA) supplemented with 10% FBS (Gibco, Grand Island, NY, USA), 100 U/mL of penicillin (Sigma, Saint Louis, MO, USA), and 100 U/mL of streptomycin (Sigma) at 28 °C in a humidified atmosphere of 5% CO_2_ incubator [32,33]. HEK293T cells were cultured in DMEM with 10% FBS at 37 °C and 5% CO_2_. pDsRed1-C1 and pCMV-enhanced GFP (pCMV-eGFP) were used for expressing hepcidin and Fpn. The full-length open reading frames of the grass carp hepcidin and Fpn were amplified by PCR with corresponding primers containing restriction enzyme adaptors (Table 1). The amplified DNA fragments were digested with restriction enzymes (*Xho* I and *Eco*R I for hepcidin; *Kpn* I and *Bam*H I for Fpn), followed by ligation into pDsRed1-C1 and pCMV-eGFP to construct pDsRed1-C1-hepcidin and pCMV-eGFP-Fpn, respectively. For transient transfection, all the vectors were transfected into L8824, FHM, or HEK293T cells by FuGENE 6 Transfection Reagent (Promega, Madison, WI, USA) according to the manufacturer’s instructions [34]. Western blot was used to confirm the expression of hepcidin and Fpn protein in FHM cells.

### 2.5. Bactericidal Activity

The colony forming unit (CFU) assay was carried out to investigate the minimum bactericidal concentration (MBC) of grass carp hepcidin peptides with following bacteria strains: *Escherichia coli* (*E. coli*, ATCC25922), *Aeromonas hydrophila* (*A. hydrophila*, provided by professor Xiaoxuan Chen [35]), *Edwardsiella ictaluri* (*E. ictaluri*, E.719), *Vibrio parahemolyticus* (*V. parahemolyticus*, NRR00440), *Staphylococcus aureus* (*S. aureus*, ATCC25923) and *Streptococcus agalactiae* (*S. agalactiae*, ATCC13813). Bacteria cells were pre-cultured in Trypticase Soy Broth (TSB) medium overnight and then transferred into fresh medium and cultured for 3 h at 37 °C. During the mid-logarithmic phase, bacteria were harvested and diluted to 1 × 10^6^ CFU/mL in the Tris-HCl buffer (10 mM Tris, 0.2% BSA, pH 7.4). Hepcidin peptides were serially diluted to the final concentrations of 0, 2, 4, 8, 16, and 32 μM in the Tris-HCl buffer and incubated with bacteria for 3 h at 28 °C. At the end of incubation, 20 μL of bacterial solution was spread on TSB agar plates and incubated overnight at 37 °C. MBC was defined as the lowest peptide concentration that killed 99.9% of the original inoculum [36]. All experimentations were tested in triplicate.

In order to measure the killing kinetics, bacterial cells were incubated with the peptide at the MBC determined by the method described above, and then sub-samples of bacterial culture were collected at the beginning of the test (0 h), followed by 5, 10, 20, 40, 80, 160, and 320 min post-incubation. Bacterial culture was plated on agar plates for colony counts.

### 2.6. Disruption of Bacterial Membrane by Hepcidin

Scanning electronic microscopy (SEM) was performed to observe the microscopic structure and morphological changes of bacterial cells after treatment with hepcidin-20 or -25, as previously described [37,38]. We incubated 5 × 10^5^ CFU of *E. coli* with Hepcidn-20, -25, or vehicle control (20 mM Tris, pH 7.0) for 2 h at 37 °C. Subsequently, samples were fixed with 2.5% glutaraldehyde overnight at 4 °C, dehydrated, vacuum dried, sputter-coated with gold, and then observed with an S-4800 field emission SEM (Hitachi, JEOL Ltd., Tokyo, Japan) at an accelerating voltage of 20 kV.

Propidium iodide (PI) dye becomes fluorescent upon binding to the intracellular nucleic acid and can therefore be used to determine the capability of amphipathic peptides to permeabilize membranes. To test whether hepcidin disrupts bacterial cell membrane, we measured uptake of PI dye after incubation of *E. coli* and hepcidin peptides. Bacterial cells (*E. coli*) were grown to mid-logarithmic phase and diluted into antibacterial buffer to 1 × 10^8^ CFU/mL. And then, the hepcidin peptides were added to the final concentrations of 16 μM and incubated for 20 min at 37 °C. Subsequently, bacteria were incubated with 3 μg/mL PI and 5 μg/mL 4′,6-diamidino-2-phenylindole (DAPI) for 5 min. After washing bacterial cells twice with Tris-HCl buffer, a small portion of the bacterial cell suspension (50 μL) was placed on a slide, mounted with a coverslip, and visualized under the fluorescence microscope. The fluorescence intensity was measured at 37 °C using wavelengths 485 nm and 520 nm filters for excitation and emission.

### 2.7. Gel Retardation Assay

We assessed whether hepcidin peptides bind to *A. hydrophila* genomic DNA (gDNA) by following a previously published method [39]. Briefly, peptides were incubated with bacterial gDNA (400 ng) at different N/P molar ratios (amino nitrogen, NH_3_^+^, of peptides and phosphate, PO_4_^−^, of genomic DNA from *A. hydrophila*) in 20 μL binding buffer (10 mM Tris-HCl, 1 mM EDTA, 1 mM DTT, 20 mM KCl, 50 μg/mL BSA, 5% glycerol, pH = 8) for 1 h at 37 °C [40]. The reaction was stopped by adding 4 μL loading buffer, and the mobility of gDNA was assessed by electrophoresis on 1% agarose gel. The intensity of the nucleic acid band was analyzed by ImageJ (ImageJ, version 1.47, NIH, Bethesda, MD, USA).

### 2.8. Cytotoxicity and Hemolytic Activity of Hepcidin

L8824 cell line was used to assess the cytotoxicity of the hepcidin-20 and -25. L8824 cell suspension (1 × 10^5^ cells/mL) was added to each well of a 96-well culture plate (100 μL per well) and incubated overnight. And then, a serial dilution of hepcidin-20 and -25 in PBS were added to each well with L8824 cells at the final concentrations of 1.5625, 3.125, 6.25, 12.5, 25, 50, and 100 μM. L8824 cells with hepcidin were cultured for 24 h under the conditions of 5% CO_2_ at 28 °C. Then, cell viability was assessed using a CCK-8 kit according to the manufacturer’s instructions [41]. Finally, the OD_490_ was measured using a microplate reader (Infinite F200; Tecan, Groedig, Austria). Negative (PBS without hepcidin peptides) and positive controls (5 μL of 10% Triton X-100) were included in the experiment.

The hemolytic activities of the peptides were also determined using grass carp erythrocytes, as previously described [42]. Briefly, approximately 3 mL of fresh peripheral blood was collected from grass carp with a heparin sodium tube, and the erythrocytes were collected by centrifugation at 500× *g* for 10 min at 4 °C. Cells were washed 3 times with PBS and resuspended in PBS to a final concentration of 2% (*v*/*v*). Subsequently, erythrocytes were incubated with the corresponding concentration of hepcidin peptides for 1 h. After centrifugation at 500× *g* for 10 min, the percentage of hemolysis was detected by measuring the OD_405_ ratio of the supernatant. The negative and positive controls were incubated with PBS or 2% Triton X-100, respectively.

### 2.9. Intracellular Labile Iron Pool (LIP) Measurement

The LIP levels were evaluated using the fluorometric assay [43]. Briefly, cells were washed twice in the PBH buffer (20 mM HEPES, 1 mg/mL BSA in PBS) and incubated with PBH buffer containing 150 nM calcein-acetomethoxy (CA-AM, Aladdin Shanghai, China) for 30 min at 28 °C. PBH buffer was used to remove residue agents and cells were washed twice with PBS. Subsequently, the cells were lysed with 0.2 × PBS, and the lysates were incubating on ice for 30 min. Cell lysates were then centrifuged at 12,000× *g* for 10 min at 4 °C to remove cellular debris. Calcein fluorescence intensity (F0) was measured at 488 nm (excitation)/518 nm (emission) in the Multiscan Spectrum microplate reader (SpectraMax i3x, Molecular Devices, Sunnyvale, CA, USA). After this initial measurement, 2,2′-bipyridine (Sigma, USA) (10 μM) was added to each well to strip iron from chelated calcein, and the fluorescence intensity (F1) was measured using the same filter combination. The change in fluorescence intensity was used to evaluate the cellular LIP.

### 2.10. Therapeutic and Preventive Effects of Hepcidin-20 and 25 in L8824 Infected with A. hydrophila

We assessed the therapeutic and preventive effects of hepcidin-20 and -25 by culturing L8824 cells with *A. hydrophila* and the hepcidin peptides. For testing therapeutic effects, L8824 cells were seed in a 24-well culture plate at a density of 5 × 10^5^ cells per well for 24 h at 28 °C. *A. hydrophila* cells cultured overnight were inoculated into fresh TSB medium and were grown for 3 h at 37 °C. Then, bacterial suspension (multiplicity of infection (MOI) = 50) in M199 medium was added to the L8824 cells and incubated for 3 h at 28 °C and 5% CO_2_. Afterwards, peptides were added to each experimental well to a final concentration of MBC and incubated for 24 h at 28 °C. The plates were examined for the presence of bacterial cytopathic effect under the microscope. For crystal violet staining, L8824 cells were seeded into 24-well plate overnight. Subsequently, 3 h (three hours) post-infection, peptides were added to each experimental well. At 24 h post-infection, the resulting cell culture medium was plated on agar plates to obtain colony count data. Then, cells were washed and fixed with 4% paraformaldehyde for 15 min at room temperature and stained with 0.05% (*w*/*v*) crystal violet (Sigma, Saint Louis, NY, USA) for 30 min, then washed with water, and drained. Finally, the plates were photographed under a light box (Bio-Rad). The clonogenic survival of irradiated cells was determined by crystal violet staining.

For testing the preventive effects of hepcidin-20 and -25, L8824 cells were seeded in a 24-well culture plate for 24 h at 28 °C. Subsequently, L8824 cells were pre-treated with M199 containing PBS, hepcidin-20 or -25 (16 μM) for 3 h, before bacterial suspension was added and incubated with cells for 24 h. Using crystal violet staining to evaluate the preventive effect of hepcidin. To investigate the effect of iron homeostasis on the L8824 cells to resist bacterial infection, FeSO_4_ and Deferoxamine (DFO) were used to increase and decrease intracellular iron content, respectively. Before bacterial infection, the L8824 cells were treated with PBS, FeSO_4_, or DFO for 3 more hours. Finally, the plates were examined for the presence of bacterial cytopathic effect under the microscope. Cell viability was monitored using crystal violet staining.

### 2.11. Protective Efficacy of Hepcidin In Vivo

*C. idella* (*n* = 500, average weight and standard deviation = 20 ± 5 g) were obtained from Hubei Bairong Improved Aquatic Seed Co, Ltd. (Huanggang, Hubei China). During this period, the water temperature of the aquariums was kept at 20 ± 5 °C, and the fish were fed twice daily with a commercial fish feed (Haid Group, China) throughout the experiments. All experiments were performed following the recommendations in the Guide for the Care and Use of Laboratory Animals of the National Institutes of Health. Similar to the in vitro experiments (Section 2.10), we performed two experiments to assess the protective effects of hepcidin in grass carp against bacterial infections.

To assess the therapeutic effects of hepcidin, *C. idella* (*n* = 30 per group in duplicate) were injected with 2 × 10^6^ CFU of *A. hydrophila*, followed by the administration of hepcidin-20/25 (0.1 and 1 μg/g hepcidin-20/-25, or equal volumes of PBS) at 3 h post-infection by intraperitoneal injection [44]. Tissue bacterial load was quantified by the dilution coated plate method. Briefly, selected fish tissues were weighed and homogenized in PBS buffer using a tissue grinder (Sigma). The tissue homogenate was serially diluted and plated onto Aeromonas Medium Base (Ryan) agar (hopebio, Qingdao China) plates for obtaining green colony counts (*A. hydrophila*). The intestines were fixed in 10% neutral-buffered formalin, and histological sections stained routinely with hematoxylin and eosin (HE) to evaluate the intestinal tissue damage. To assess whether the iron in the tissue was overloaded or exhausted, iron in hepatopancreas were detected by Prussian blue staining was performed as described [25]. Stained sections were observed under bright field illumination using a Nikon Eclipse E100 microscope equipped with a digital camera.

For the preventive experiments, *C. idella* (*n* = 30 per group in duplicate) received a single dose of 0.1 μg/g of hepcidin-20/-25, 1 μg/g hepcidin-20/-25, or equal volumes of PBS by intraperitoneal injection. Twenty-four hours post-peptides injection, *C. idella* were received a lethal dose of 2 × 10^6^ CFU of *A. hydrophila* [44]. The method of treatment group was used to detect the bacterial load in the tissues.

### 2.12. Statistical Analysis

Statistical analysis was performed using Graphpad Prism 7.0 software (GraphPad, San Diego, CA, USA). The independent *t*-test was used for comparing means between groups in each data-set. *p*-value < 0.05 was considered a statistically significant difference [32].

## 3. Results

### 3.1. Grass Carp Hepcidin Is a Cationic and Amphipathic Protein that Can Be Induced by Immunostimulants

**Figure 1:**
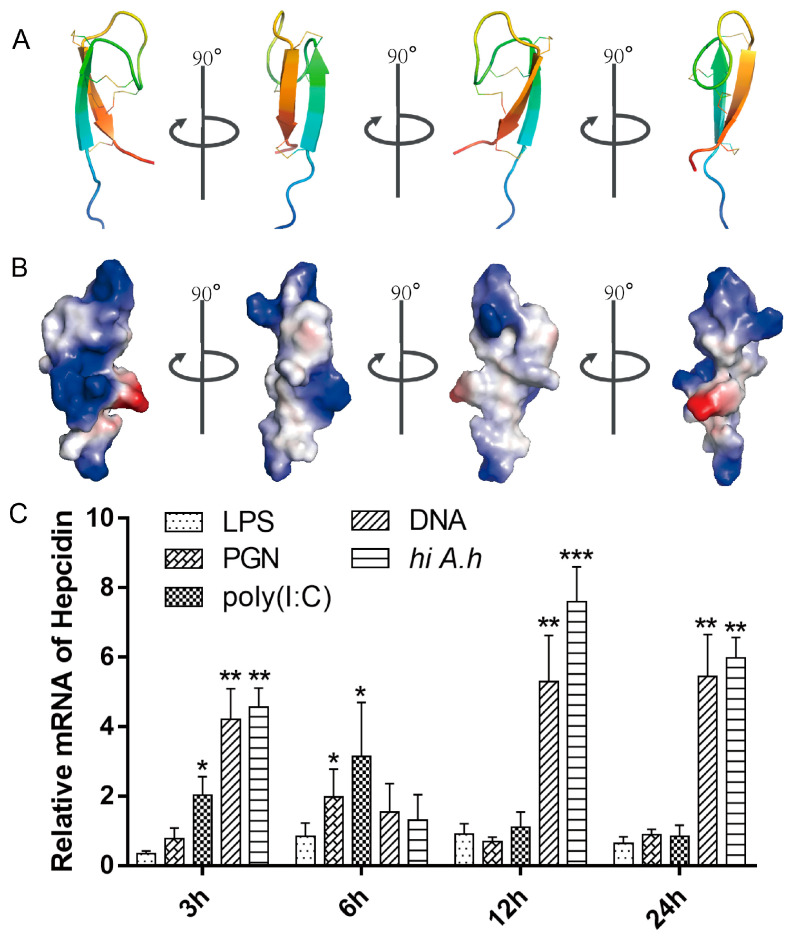
Hepcidin is a cationic amphipathic protein that is induced by heat-inactivated bacteria or bacterial DNA. (**A**) Protein ribbon of hepcidin obtained by homology modelling method. (**B**) Colour-coded electrostatic potentials mapped onto the surfaces of hepcidin. Regions of positive charges are in blue, and negative charges are in red, and hydrophobic charges are in white. (**C**) L8824 cells were seed in 12-well plates for 24 h adherence. Subsequently, cells were stimulated with 2 μg/mL LPS, PGN, poly(I:C), *A. hydrophila* DNA, or heat-inactivated *A. hydrophila* (*hi A. h*) for 3 h, 6 h, 12 h and 24 h and the expression of hepcidin was measured by qRT-PCR.

Hepcidin is composed of 25 amino acid residues, with a molecular weight of 2.98 kDa. The deduced amino acid sequence of grass carp hepcidin showed approximately 44% identical and 55% similarity to those of humans. Prediction of the tertiary structure revealed that the grass carp hepcidin consists of N-terminal (5-AA) and β-sheet (20-AA), which can lead to hydrophilic cluster formation and surface exposure of the cationic residues (Figure 1A,B). On the opposite side of this hydrophilic cluster, we observed a hydrophobic patch composed of several hydrophobic side chains (Leucine 4, Leucine 6, Tyrosine 9, Tyrosine 21, and Phenylalanine 25). The predominance of polar (cationic) residues on one side of the molecule and hydrophobic amino acids on the opposite side indicated that hepcidin is a cationic amphipathic protein. This is consistent with the characteristics of antimicrobial peptides [45].

To elucidate which factor induces hepcidin, the *hi A. h* and four representative pathogen-associated molecular patterns (LPS, PGN, poly (I:C) and *A. hydrophila* gDNA) used to stimulate the L8824 cells derived from grass carp. As shown in Figure 1C, the mRNA expression of hepcidin was significantly increased in the cells treated with *A. hydrophila* gDNA and *hi A. h*. These results indicated that hepcidin could be strongly induced by bacterial gDNA.

### 3.2. In Vitro Antimicrobial Activity, Cytotoxicity, and Hemolytic Toxicity

**Figure 2:**
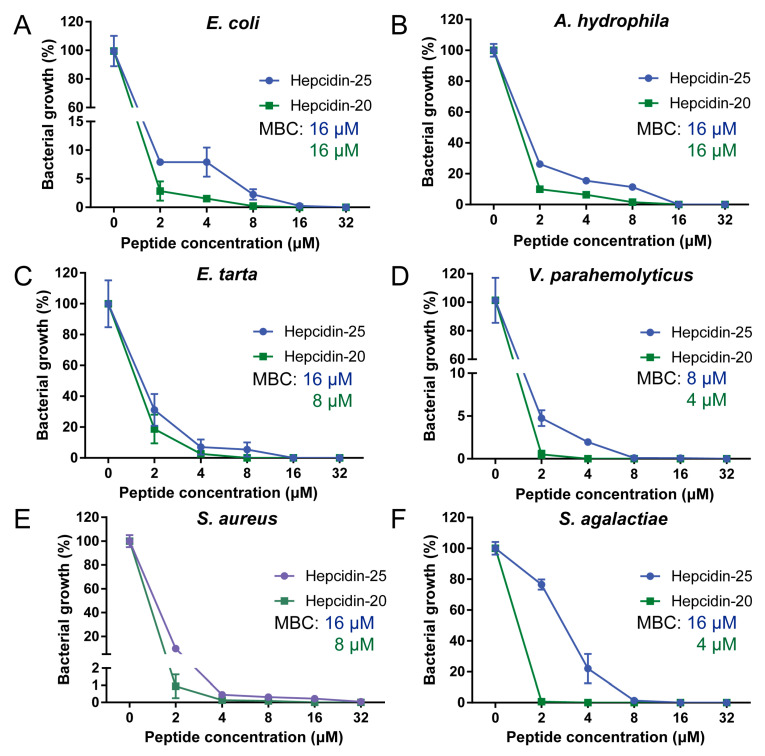
Antibacterial activities of hepcidin-20 and 25 against Gram-positive and Gram-negative bacteria in vitro. Antibacterial activities of hepcidin-25 and -20 against *E. coli* (**A**), *A. hydrophila* (**B**), *E. ictaluri* (**C**), *V. parahemolyticus* (**D**), *S. aureus* (**E**) and *S. agalactiae* (**F**). Bactericidal activity was detected by CFU assay as described in “Materials and Methods”. Error bars indicated SE for triplicate determinations.

**Figure 3:**
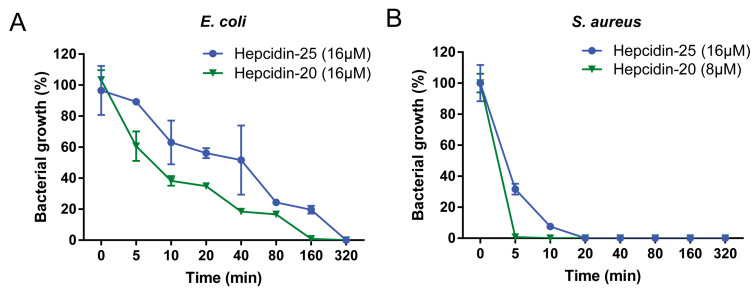
Bacteria killing kinetics. *E. coli* (**A**) and *S. aureus* (**B**) were treated with hepcidin-20 and -25 (1 × MBC) at 28 °C. Subsequently, the viability of the bacteria cells was measured by CFU counts at the indicated time point. Samples were measured in triplicates.

Using the CFU assay, the MBC of hepcidin peptides in TSB medium was carried out to detected. As shown in Figure 2, hepcidin-20 and -25 displayed a broad spectrum of antibacterial activities against both Gram-negative (*E. coli*, *A. hydrophila*, *E. ictaluri*, and *V. parahemolyticus*) and Gram-positive bacteria (*S. aureus* and *S. agalactiae*). The antibacterial activities of hepcidin-20 were 2 to 4-fold higher than that of hepcidin-25, which MBC values for Gram-positive and Gram-negative bacteria ranging from 2 to 8 μM. The data from kinetics of the hepcidin-20 and -25 revealed that *S. aureus* was killed within 20 min of incubation with hepcidin-20 (8 μM) or hepcidin-25 (16 μM) (Figure 3B). In contrast, it took over 160 min to kill *E. coli* under the same test condition (Figure 3A).

**Figure 4:**
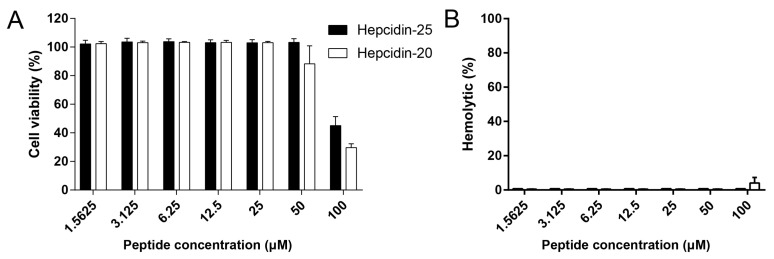
Cytotoxicity and hemolytic assay of hepcidin-20 and -25. (**A**) L8824 cells were incubated with varying concentrations (0 to 100 μM) of peptides for 24 h at 28 °C. Cell viability was assessed using a CCK-8 kit. (**B**) Hemolytic activities of peptides (0 to 100 μM) were detected with 2% grass carp erythrocytes for 1 h at 28 °C. 2% Triton X-100 was used as a positive control.

Hepcidin-20 and -25 showed cytotoxicity at the highest concentration tested (100 μM) and reduced cell viability to approximately 40% and 30%, respectively (Figure 4A). However, no cytotoxicity was observed at low concentrations below 25 μM (Figure 4A). Similarly, the hepcidin peptides showed very little hemolytic activity against erythrocytes from grass carp (Figure 4B).

### 3.3. Hepcidin Kills Bacteria by Directly Disrupting the Bacterial Cell Membrane and Binding Bacterial gDNA

**Figure 5:**
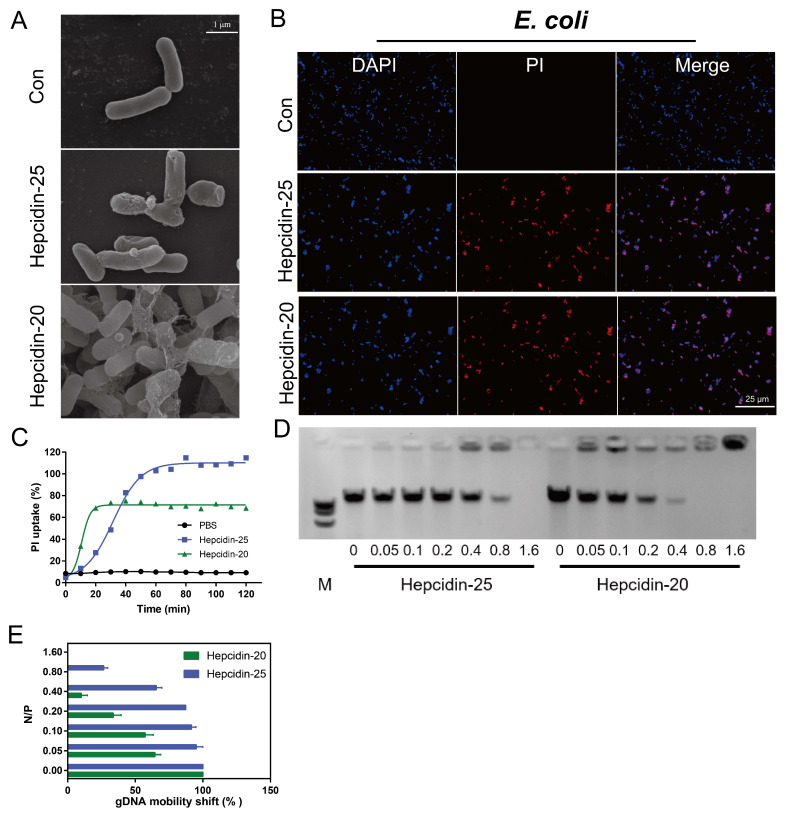
Sterilization mechanism of hepcidin-20 and -25. (**A**) Scanning electron microscopy of *E. coli* cultured with or without hepcidin-20/25 (16 μM) for 2 h at 37 °C. (**B**) PI update by *E. coli* after incubated with 16 μM hepcidin-20 or hepcidin-25 for 2 h at 37 °C. Bacteria were stained with DAPI (blue) or PI (red) and imaged using a confocal microscope. (**C**) Using the PBS as the control, PI uptake of *E. coli* by hepcidin-20 (green) and -25 (blue). (**D**) Gel shift assay of bacterial gDNA mixed with increasing concentrations of hepcidin-25 (left) and -20 (right). The abscissa represents the N/P molar ratio (amino nitrogen (NH_3_^+^) of peptides/phosphate (PO_4_^−^) of DNA). (**E**) ImageJ was used to analyze the intensity of nucleic acid bands in gel shift assay. The abscissa represents the N/P molar ratio and the ordinate represents the gDNA.

Scanning electron microscopy demonstrated that bacterial cells (*E. coli*) membranes were disrupted by both hepcidin-20 and -25 (Figure 5A). However, the mechanism of membrane disruption seems to be different between hepcidin-20 and -25: hepcidin-25 caused cell membrane damage while hepcidin-20 caused *E. coli* aggregation and the net-like structure surrounding bacteria. When *E. coli* (Figure 5B) was incubated with hepcidin-20 and -25, we observed a significant increase in the PI fluorescence signal with an increase of hepcidin in a dose-dependent manner (Figure 5C), indicating that these peptides permeabilize the bacterial membrane and thereby disrupt the integrity of the bacterial membrane.

As research showed that positive surface charge from antimicrobial peptides had been shown to form complexes with bacterial gDNA [39], we tested whether hepcidin-20 and -25 bind gDNA during bacterial killing. The data from the hepcidin binding experiment indicated that the intensity of the gDNA band gradually decreased as the ratio of hepcidin peptide per bacterial gDNA increased (Figure 5D). This trend was particularly significant in hepcidin-20. Quantification of band intensities using the grayscale image of the agarose gel showed that hepcidin-20 was approaching the saturation of the gDNA binding sites at the ratio of 0.4, while hepcidin-25 was beginning to bind at that point. We found that a ratio of hepcidin-20/gDNA = 0.4 was sufficient to induce the DNA mobility shift. In contrast, a hepcidin-25/gDNA = 0.4 has only just begun to induce the DNA mobility shift. (Figure 5E). These results demonstrated that both hepcidin-20 and -25 bind to bacterial gDNA in a dose-dependent manner, however the affinity to the bacterial gDNA was different between the two peptides.

### 3.4. Antibacterial Activities of the Peptides in L8824 Cells

**Figure 6:**
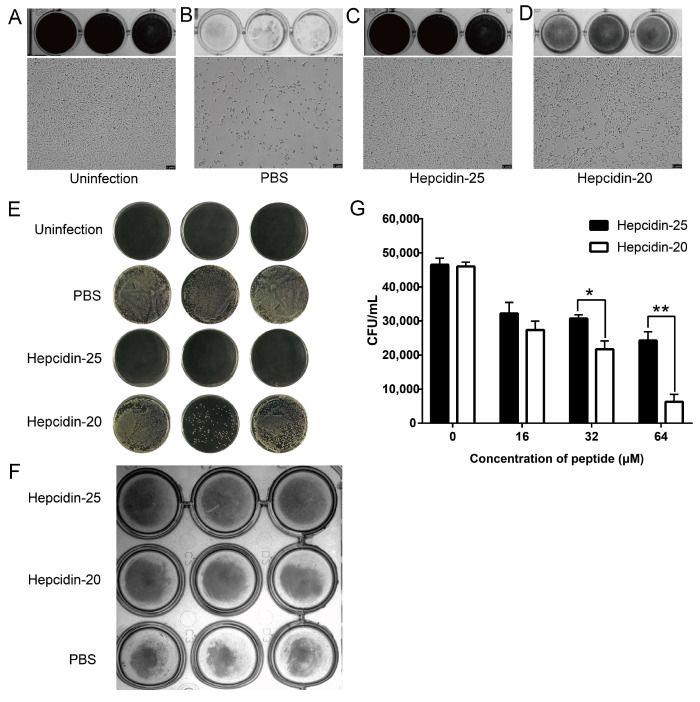
Therapeutic and preventive effects of hepcidin-20 and 25 in L8824-infected bacteria. (**A**–**D**) Bacterial suspension in M199 was incubated with L8824 cells for 3 h and then treated with PBS (**B**), hepcidin-20 (**C**), or -25 (**D**) (1 × MBC 16 μM) at 28 °C for 24 h. Cell density was determined using phase-contrast microscopy and crystal violet staining. Uninfected cells (**A**) were used as blank control. (**E**) After completing the above processing, the resulting cell culture medium was plated on agar plates for colony counts. (**F**) L8824 cells were treated with PBS, hepcidin-20, or -25 for 3 h, and then bacterial suspension in M199 was incubated with cells for 24 h. Subsequently, cell density was determined using phase-contrast microscopy and crystal violet staining. (**G**) The CFU assay was employed to investigate the antibacterial activity of hepcidin-20 and -25 in the M199 medium. Image shown were representative of three independent experiments, * *p* < 0.05; ** *p* < 0.01.

Based on the above two experiments, we further investigated the therapeutic and protective effects of hepcidin-20 and -25 in L8824 cells infected with *A. hydrophila*. Crystal violet staining and microscopic observations showed that a large number of cells died during PBS treatment. However, a significant protection was observed when cells were treated with hepcidin-20 and -25 at 3 h post-infection of *A. hydrophila* (Figure 6A–D). In addition, the plate count results showed that both peptides, particularly hepcidin-25, significantly reduced the bacterial cell numbers in the M199 medium (Figure 6E). Similarly, protection was observed by treating L8824 cells with hepcidin prior to bacterial infection (Figure 6F). However, cell survival rate in hepcidin pre-treatment was not as high as post-treatment. Although a relatively high level of protection was observed in post-treated target cells, modest inhibition of infection was also seen when peptides were added to cultures up to 3 h before infection. Surprisingly, contrary to MBC test results, hepcidin-25 showed a better antibacterial effect than hepcidin-20 in L8824 cells. However, many studies have shown that culture condition changes have a great impact on the antimicrobial activity of the peptide. To rule out this possibility, we examined the antibacterial activities of the peptides in the M199 medium. However, the results showed that the change in antibacterial buffer was not the reason for subverting the antimicrobial ability of the two peptides (Figure 6G).

### 3.5. Hepcidin-Fpn Pathway Regulates Iron Homeostasis and Promotes Antibacterial Immunity

To evaluate whether the hepcidin and Fpn could regulate the iron metabolism in the teleost, we established the L8824 cell line overexpressing *C. idella* hepcidin and Fpn and assess the mRNA level of iron metabolism-related genes and intracellular LIP content. We confirmed that the mRNA and protein levels of hepcidin and Fpn were markedly overexpressed in the L8824 cell line (Supplementary Figure S2B). Over-expression of hepcidin gene in L8824 cells increased the intracellular LIP contents and the expression of Ferritin gene (Supplementary Figure S2C,E) while an opposite trend was observed by over-expression of Fpn (Supplementary Figure S2F). Hepcidin triggers Fpn polyubiquitination and induces its endocytosis to prevent the efflux of intracellular iron [6,11,12,13].

**Figure 7:**
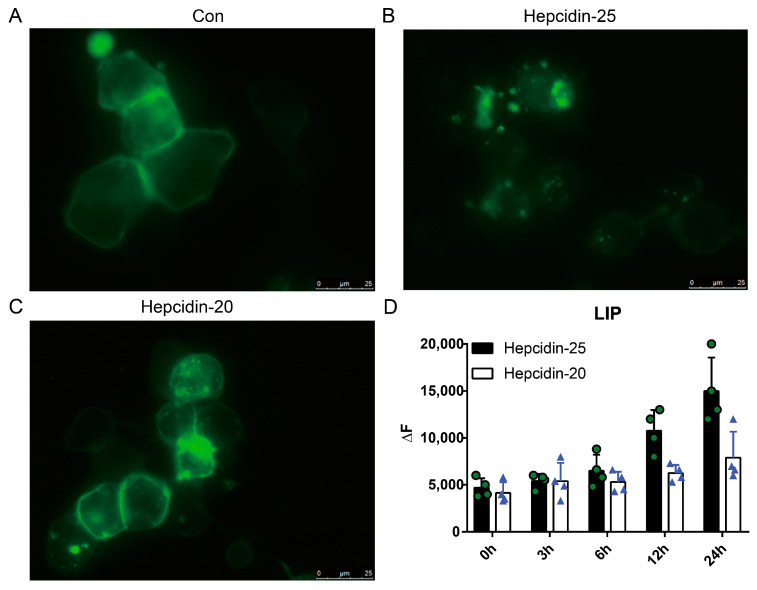
The effects of hepcidin-20 and 25 on iron homeostasis. HEK293T cells were seeded in 6-well plates 24 h before transfection with Fpn-eGFP. 24 h after transfection, cycloheximide (75 μg/mL) was added for 3 h, and then cells were incubated with PBS (**A**), hepcidin-20 (1 μM) (**B**) or hepcidin-25 (1 μM) (**C**) for at least 24 h. (**D**) L8824 cells were seed in 12-well plates for 24 h. Next, cells were stimulated with hepcidin-25 or hepcidin-20, and the LIP content was detected by fluorometric assay.

Subsequently, we tested the ability of these peptides to internalize and degrade Fpn. Our result showed that only stimulated with hepcidin-25 significantly attenuated the fluorescence intensity of Fpn (Figure 7A–C), which confirmed the involvement of peptide in cellular iron metabolism. Hepcidin-25 also enhanced the intracellular LIP contents (Figure 7D).

To investigate the effect of iron homeostasis on the L8824 cells to resist bacterial infection, we used FeSO_4_ or DFO to model iron overload and iron deficiency, respectively. The results showed that FeSO_4_ and DFO treatment increased or decreased the LIP contents, respectively (Figure S3A). Subsequently, we found that increasing the intracellular LIP contents protected cells from bacterial infection to a certain degree (Supplementary Figure S3B,C). However, a combination of FeSO_4_ and DFO eliminates the protection of cells against bacterial infection (Supplementary Figure S3D). Additionally, we found that *A. hydrophila* gDNA increased the intracellular LIP content and expressions of Ferritin, IL-6, iNOS in L8824 cells (Supplementary Figure S4). These results showed that iron metabolism and antimicrobial immunity were inseparable.

### 3.6. Hepcidin Is a Prophylactic and Therapeutic Agent against A. hydrophila Infection in C. idella

**Figure 8:**
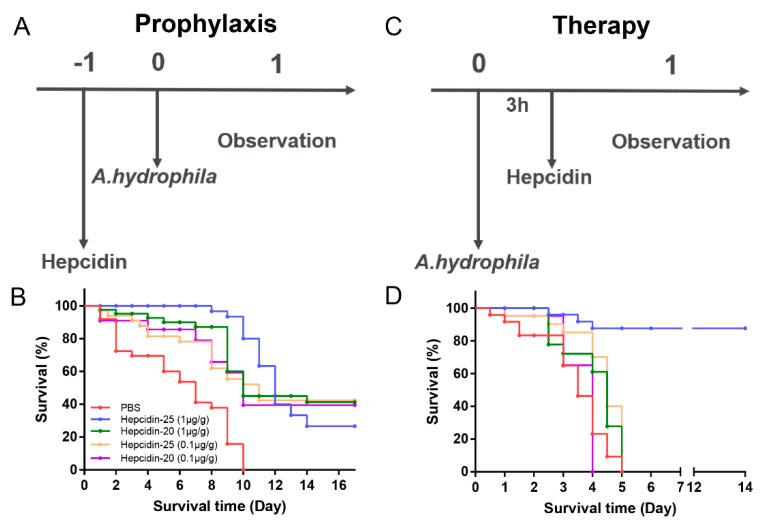
Protective efficacy of hepcidin in the grass carp model of intestinal tract infection. (**A**) Schematic of the *A. hydrophila*-infected prophylactic model. (**B**) Preventive efficacy of hepcidin in the *A. hydrophila*-infected prophylactic model. *C. idella* were received intraperitoneal injection at a single dose of 0.1 μg/g hepcidin-20/25, 1 μg/g hepcidin-20/-25, or equal volumes of PBS (*n* = 30). Twenty-four hours later, *C. idella* have received a lethal dose of 1 × 10^6^ CFU of *A. hydrophila*. (**C**) Schematic of the *A. hydrophila*-infected therapeutic model. (**D**) Therapeutic efficacy of hepcidin in *A. hydrophila*-infected therapeutic model. *C. idella* were injected intraperitoneally with a lethal dose of 1 × 10^6^ CFU of *A. hydrophila* and then received a single dose of 0.1 μg/g hepcidin-20/-25, 1 μg/g hepcidin-20/-25, or equal volumes of PBS (*n* = 30) at 3 h post-infection. The mortality in each group was recorded for at least 14 days.

We established an infection model of *A. hydrophila* in grass carp (Supplementary Figure S5). Grass carp infected with *A. hydrophila* exhibited ulcer on fish skin, accumulation of ascites, the intestinal bleeding, and the bacterial overload in the intestines and spleens (Supplementary Figure S5C). As hepcidin maintains iron homeostasis and bacterial killing, the preventive efficiency of hepcidin against bacterial infection was tested in the grass carp model of enteritis. As shown in Figure 8A, *C. idella* received a single intraperitoneal injection of peptides 24 h before the *A. hydrophila* infection (1 × 10^7^ CFU/mL, 100 μL). The survival rate of *C. idella* treated with 1 μg/g hepcidin-25 prior to bacterial inoculation (preventive effects) was 100% in the first seven days. Fish receiving 1 μg/g hepcidin started to die at Day 9. At the end of the experiment, all the experimental groups showed different degrees of protection effects than the control groups (Figure 8B). About 40% of fish survived by administration of hepcidin, while the survival rate of the control group is 0%.

For testing the therapeutic effects, *C. idella* received a single intraperitoneal injection of peptides three hours after infection. (Figure 8C). As shown in Figure 8D, a survival rate of 83.3% (25 out of 30) at day 14 after infection was observed in grass carp administrated with 1 μg/g hepcidin-25, whereas all fish died in day 5 were observed among the PBS, 0.1 μg/g hepcidin-25-, 0.1 μg/g hepcidin-20-, and 1 μg/g hepcidin-20-treated groups.

### 3.7. Hepcidin Affects Iron Homeostasis and Tissue Inflammation

**Figure 9:**
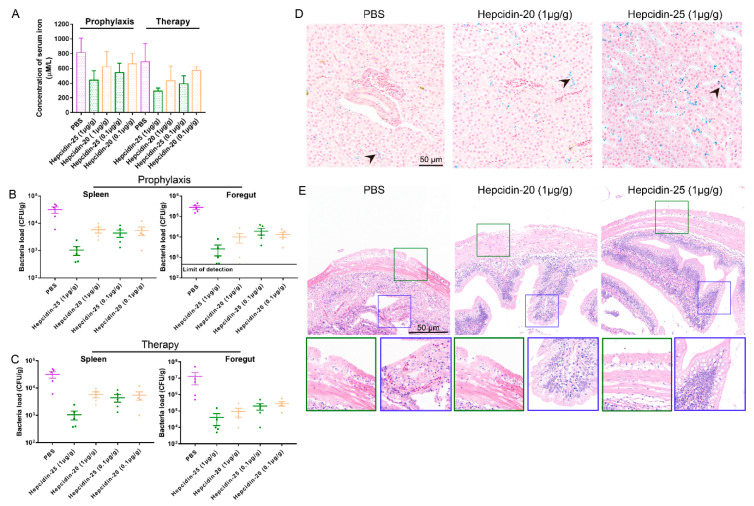
The effect of hepcidin on iron homeostasis and bacterial loads of spleen and foregut infected with *A. hydrophila*. (**A**) The serum iron assay kit quantified the iron content in serum. (**B**,**C**) At 3 days post-infection, bacterial loads in spleen and foregut of grass carp infected with *A. hydrophila* in prophylaxis (**B**) and therapy (**C**) groups were detected, (*n* = 5). (**D**) At 3 days post-infection, using the PBS group as the control, the iron content in hepatopancreas was stained by Prussian blue, bar = 50 μm. Arrowheads in the image show the iron content in hepatopancreas. (**E**) At 3 days post-infection, the intestinal tissue sections in the 1 μg/g hepcidin-20 and -25 group were observed, serous membrane (green) and intestinal villi (blue).

To further examine the effects of hepcidin in reducing the virulence of *A. hydrophila* on the grass carp, we evaluated pathological changes and bacterial loads in organs after infection. Iron contents in serum were also measured. The data from colony count and histopathology demonstrated that the administration of hepcidin-20 and -25 in both prophylactic and therapeutic groups significantly decreased the *A. hydrophila* loads in both foregut and spleen (Figure 9B,C). As shown in Figure 9A, serum iron concentration was reduced 50% by the therapeutic effect of 1 μg/g hepcidin-25, while hepcidin-20 had a weaker effect on serum iron.

Since the liver is the main organ of iron metabolism and *A. hydrophila* causes serious enteritis, we detected the iron content in hepatopancreas and observed the intestinal tissue sections in the 1 μg/g hepcidin-20 and -25 groups at day 3 post-infection. Subsequent Prussian blue staining showed that iron content in hepatopancreas in 1 μg/g Hepciidin-25 group increased compared to the PBS on day 3 post-infection (Figure 9D). As shown in Figure 9E, the intestinal villi and serous membrane bleeding were significantly congested in the PBS group because of bacterial infection. However, there was no significant bleeding observed in 1 μg/g hepcidin-20 and -25 treated groups. In addition, treatment with 1 μg/g hepcidin after infection was accompanied by an increase in the number of goblet cells in the intestinal villi (Figure 9E). These goblet cells protect the epithelium from dehydration, physical abrasion, and commensal and invading microorganisms [46,47,48].

## 4. Discussion

Hepcidin is a small, cysteine-rich antimicrobial peptide that plays an important role in controlling bacterial infectious diseases. It has been reported that the hamp type-1 in fish maintains iron homeostasis by regulating iron uptake and distribution, such as largemouth bass (*Micropterus salmoides*) and turbot (*Scophthalmus maximus*) [49,50]. Unlike most fish species and some mammals have multiple genes of hepcidin [16,22], only one type of hepcidin has been found in grass carp so far. The hepcidin of grass carp was thought to belong to type-1 based on its sequence characteristics and iron regulation activities [25]. To our surprise, we noted that the structural features (amphiphilic structure and net positive charge) of hepcidin in grass carp were consistent with an antimicrobial peptide, which led us to generate a hypothesis that grass carp hamp1 has both iron regulation (type 1) and antibacterial activities (type 2) (Figure 1).

As expected, grass carp hepcidin lowered intracellular iron concentration by overexpression (Figure S2), supporting the finding from our previous study [25]. Induced Fpn internalization and degradation are key events in maintaining iron homeostasis, and the data from this study demonstrated that grass carp hepcidin (hamp type-1) induced Fpn endocytosis and thereby involved in regulation of iron homeostasis (Figure 7A,B). This is consistent with the results in human [51,52]. Hepcidin-induced hypoferremia has been proposed as an important host defense mechanism [51,53]. Experimental studies have shown that iron is a principal element required for bacterial growth, and plays a key role in many fundamental biological processes [54]. Additionally, the growth of pathogens in their hosts is critically dependent on the pathogens’ ability to capture and utilize iron. The hepcidin restricts the bioavailability of iron and effectively starve the microbes [53].

The data from bactericidal activity tests revealed that grass carp hamp type-1 has antibacterial activity supporting the hypothesis that grass carp hamp type-1 has both iron regulation and antibacterial activities (Figure 2). This is contrary to other fish species having different hepcidin type with distinct biological functions. The antibacterial function of hepcidin is mainly concentrated in hamp type-2 [16,21,22,23,55,56,57]. Hamp type-1 (hamp type-2 isoform) isolated from other fish species, such as Japanese flounder (*Paralichthys olivaceus*), mudskipper (*Boleophthalmus pectinirostris*) and European sea bass (*Dicentrarchus labrax*), do not have such antibacterial activities [20,21,22]. Although the subsequent studies have shown that the hamp type-1 of turbot and mudskipper (*Boleophthalmus pectinirostris*) have antibacterial activity, but its activity was very weak and did not promote the host defense against bacterial infections [16,22]. Our finding demonstrated that the grass carp has only one hepcidin, which is involved in both iron homeostasis regulation and bacteria-killing. Although speculative, grass carp hepcidin could be an ancestral form of type-1 and 2 found isolated in other fish species.

Apparently, the grass carp hepcidin inhibits growth of bacterial cells by two mechanisms: disrupting bacterial cell membrane and interacting with bacterial gDNA (Figure 5A,D). Many antimicrobial peptides have been found to kill bacteria by binding to DNA, such as pleurocidin [58], NK-18 [59] and piscidin [60]. Studies have shown that human and fish hepcidin kills a particular group of bacteria by hydrolysing bacterial gDNA, however do not induce any loss of bacterial membrane integrity [61,62]. Different from previous membrane-independent results [63,64], our results demonstrated that hepcidin mature peptide (hepcidin-25) and truncated peptide (hepcidin-20) exhibited apparent bactericidal activities against both Gram-positive and Gram-negative bacteria in a dose-dependent manner in part through membrane rupture and binding to gDNA. Disruption of bacterial cell membranes by grass carp hepcidin is a new finding since other hepcidin does not have such a function. Antimicrobial peptide NK-18 kills bacteria by disturbing the bacterial membrane and binding to DNA [59]. The double target strategy of these peptides makes it difficult for bacteria to develop resistance [58]. Thus, hepcidn could be a candidate agent to treat bacterial infection.

Our results show that hepcidin promotes antimicrobial defense via direct microbial killing and regulating iron metabolism in grass carp. Previous studies have shown that another mechanism by which hepcidin may potentially exert strong antimicrobial effects in vivo is through synergistic interactions with other inducible acute phase response proteins and/or constitutively expressed antimicrobial compounds in the tissues, such as moronecidin [54]. However, whether there are some hepcidin synergistic molecules in grass carp deserves further study. The data that grass carp hepcidin inhibits bacterial growth directly (disrupt the integrity of bacterial cell membrane or aggregate bacterial cells and subsequently hydrolyse bacterial gDNA) and indirectly (lower iron concentration in fish cells or body) suggest that grass carp hepcidin can be possibly used as a method for controlling bacterial infection in aquaculture. As expected, prophylactic or therapeutic administration of hepcidin increased the survival rate of grass carp that were experimentally infected with a lethal dose of *A. hydrophila* (Figure 8), which was accompanied by a lower bacterial cell number in the foregut and spleen of the fish (Figure 9). The therapeutic effect of grass carp hepcidin is better than its preventive effect, especially the injection of hepcidin-25 (1 μg/g) with about 85% survival rate after grass carp infection of bacterial. The survival rate of *C. idella* treated with 1 μg/g hepcidin-25 prior to bacterial inoculation (preventive effects) was 100% in the first seven days. Fish receiving 1 μg/g hepcidin started to die at Day 9. Although speculative, this may be because the immune response caused by the injection of hepcidin can promote antibacterial immunity, but it cannot completely eliminate the pathogenic bacteria and leads to death after nine days.

To elucidate the role of five amino acid residues at the N-terminus of grass carp hepcidin, we chemically synthesized hepcidin-20 and -25 and evaluated differences of their biological activities. The most significant difference between hepcidin-20 and -25 was that hepcidin-20, lacking five amino acid residues at the N-terminus, showed a lower protective effect against a lethal dose of *A. hydrophila* injection than hepcidin-25 in the in vivo challenge test (Figure 8). Unlike hepcidin-25, which showed both antibacterial and iron regulation functions, hepcidin-20 only showed bactericidal function as demonstrated by in vitro study (Figure 2 and Figure 6). Hepcidin-20 has almost lost the ability to internalize Fpn (Figure 7A–C). Although hepcidin-20 reduced bacteria load in tissues, there was no significant effect on serum iron and liver iron levels (Figure 9A,D). All our data suggest that the five amino acid residues at the N-terminus of the hepcidin are a key site for controlling iron concentration in serum and liver in grass carp. In humans, hepcidin is mainly synthesized in the liver as an 84 amino acid pro-peptide. Subsequently, the pro-peptide is broken down into smaller, mature peptides of 25 amino acids, which are secreted in plasma. The mature peptide can undergo further truncation in its N-terminal, resulting in the production of two smaller isoforms, hepcidin-20 and -22. The functional studies have shown that the two truncated isoforms of human hepcidin-20 and -22 almost completely lost their ability to interact with Fpn [1,63,65,66]. Lowering iron concentration is not always needed to fight against bacterial infection. This may be because an excessive lack of iron can lead to iron-deficiency anemia that can be critical to a host in some cases. [67]. Currently, it is unknown whether grass carp hepcidin-25 undergo further truncation and produce shorter forms such as hepcidin-20, however it likely to happen given the fact that a thermolysin recognition site is present in the amino acid sequence of hepcidin-25 (data not shown). Because the website (https://web.expasy.org/peptide_mass/) predicts that thermolysin can cut hepcidin-25 into hepcidin-20. Further study is needed to identify whether hepcidin-20 is produced in grass carp.

In summary, hepcidin plays an important role in host defense against siderophile bacteria such as *A. hydrophila* by direct killing of bacteria and reducing the available iron concentration. These effects prevent the rapid growth of *A. hydrophila* so that other innate immune mechanisms are sufficient to control the infection. Pointing to potential therapeutic applications, pre- or post-infection administration of hepcidin agonists increased their resistance to *A. hydrophila* infection and protected them from consequent mortality.

## 5. Conclusions

Hepcidin is an antimicrobial peptide and a regulator of iron homeostasis which has two isoforms in most fishes and some mammals. Previous studies have reported that the two hepcidin isoforms have different roles. Hamp type-1 plays a regulatory role in iron metabolism and hamp type-2 mostly performs an antimicrobial role. However, in this study, we found that *Ctenopharyngodon idella* (*C. idella*) have only one hepcidin isoform (hamp type-1), which showed both broad-spectrum antibacterial and iron regulatory functions. *C. idella* hepcidin mature peptide (hepcidin-25) and truncated peptide (hepcidin-20) exhibited bactericidal activities against both Gram-positive and Gram-negative bacteria in a dose-dependent manner in part through membrane rupture and binding to bacterial genomic DNA. In addition, a comparison between hepcidin-20 and -25 suggests that the N terminal 5 amino acids play a critical role in reducing iron content in fish serum. These effects prevent the rapid growth of *A. hydrophila* so that other innate immune mechanisms are sufficient to control the infection. Pointing to potential therapeutic applications, pre- or post-infection administration of hepcidin agonists increased their resistance to *A. hydrophila* infection and protected them from consequent mortality.

## Figures and Tables

**Table 1:** Primers used in the experiment.

Gene	Primer Name	Forward Primer (5′ → 3′)	Primer Name	Reverse Primer (5′ → 3′)
EF1α	EF125	CGCCAGTGTTGCCTTCGT	ER126	CGCTCAATCTTCCATCCCTT
Hepcidin	HeF91	TGAAACACCACAGCAGAACGA	HeR92	CAGCCTTTGTTACGACAGCAGTT
Ferroportin	Ferroportin-F	CACAGATTCAGACAAATGCCA	Ferroportin-R	TGTCGTTCGGTTCCCATTT
Ferritin	Ferritin-F	TCCTGTGCTTCGTGCGTGT	Ferritin-R	ACCTTCAGTCCGTCCTCGTG
IL-6	IL-6-F	ACAGCAGAATGGGGGAGTTATC	IL-6-R	CTCGCAGAGTCTTGACATCCTT
iNOS	iNOS-F	CGAATACGCAATGGGAGAAC	iNOS-R	GTGTCATAGCCTTTGGAGTCATAA
TLR9	TLR9-F	CAGTTGCGTTATCTCGGGGT	TLR9-R	TGGCATGAGCGAAGGTCAAT
TLR21	TLR21-F	GTCAGAGCTTCCTTCGCAGT	TLR21-R	GGACAGTTGCTGCTTGGGTA

## Supplementary Materials

The following are available online at https://www.mdpi.com/2218-273X/10/6/825/s1, Figure S1: Purity analysis of hepcidin-20 and -25, Figure S2: The hepcidin-Fpn axis controls the iron content in L8824 cells, Figure S3: Cellular iron homeostasis affects the ability of L8824 cells to resist bacterial infection, Figure S4: Effect of bacterial gDNA on iron homeostasis and immune regulation in L8824 cells, Figure S5: Grass carp model of intestinal tract infection established by *A. hydrophila* strains.
