# Supplementary material for: Hamp Type-1 Promotes Antimicrobial Defense via Direct Microbial Killing and Regulating Iron Metabolism in Grass Carp (Ctenopharyngodon idella)
Source: Biomolecules. 2020 May 28;10(6):825. doi: 10.3390/biom10060825 (PMC7356000; doi:10.3390/biom10060825)
Supplement: Supplementary file 1 [file biomolecules-10-00825-s001.pdf]

Fig.S1

A

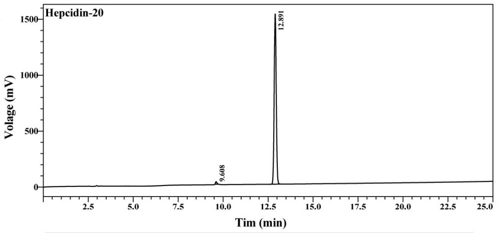

Peak table

Peak#	Ret. time	Area	Height	Area %
1	9.608	129976	23044	1.057
2	12.891	12168735	1520833	98.943
Total		12298711	1543877	100.000

B

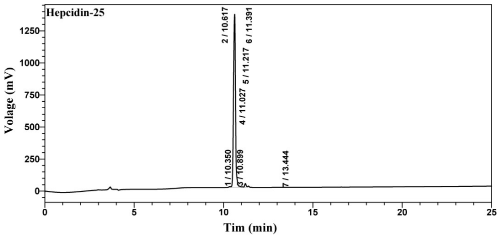

Peak table

Peak#	Ret. time	Area	Height	Area %
1	10.350	48494	7745	0.450
2	10.617	10420929	1347980	96.626
3	10.899	57510	9567	0.533
4	11.027	18626	3617	0.173
5	11.217	178514	29160	1.655
6	11.391	55858	9653	0.518
7	13.444	4887	788	0.045
Total		10784818	1408510	100

Figure.S1. Purity analysis of hepcidin-20 and -25. The purified hepcidin-20 **(A)** and -25 **(B)** obtained following preparative RP-HPLC.

Fig.S2

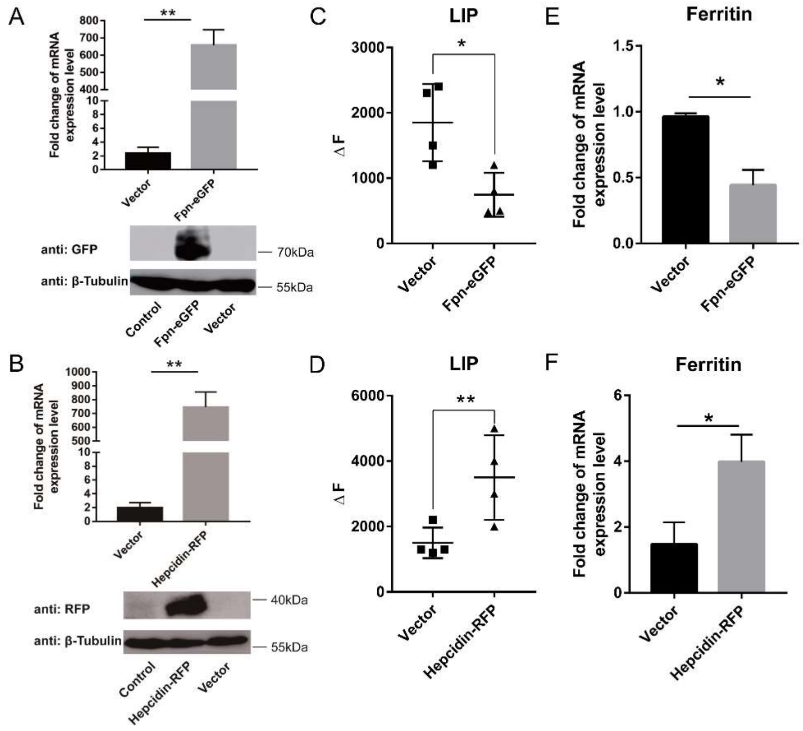

Figure.S2. The hepcidin-Fpn axis controls the iron content in L8824 cells. (A and B) pFpn-eGFP, pHepcidin-RFP, and empty vector transiently transfected L8824 cells were seeded in 12-well plates. qRT-PCR and western blot (WB) were used to assess the efficiency of Fpn and hepcidin overexpression. **(C and D)** Intracellular labile 219 iron pool (LIP) levels in Fpn and hepcidin overexpression samples were detected by fluorometric assay. Calcein (CA) fluorescence intensity was measured in the Multiscan Spectrum microplate reader. **(E and F)** The Ferritin expressions were determined at 24 h after cell transfection of plasmid by qRT-PCR. Results were expressed as mean ± SD (n=4), $^{*}P< 0.05$; $^{**}P < 0.01$.

Fig.S3

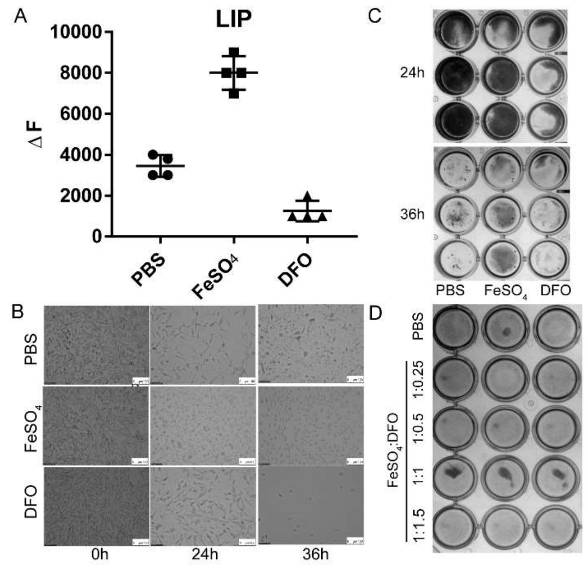

Figure.S3. Cellular iron homeostasis affects the ability of L8824 cells to resist bacterial infection. (A) Cells were seed in 12-well plates for 24 h and then treated with PBS, $FeSO_4$, or DFO for 3 more hours. The LIP levels were detected by fluorometric assay, and the CA fluorescence intensity was measured in the Multiscan Spectrum microplate reader. After stimulated with PBS, $FeSO_4$, or DFO, the cells were incubated with *A. hydrophila* (5×10^6 CFU/mL, 500 μL) for 24 h. Cell density was determined using phase-contrast microscopy **(B)** and crystal violet staining **(C)**. **(D)** Mix $FeSO_4$ and DFO in different proportions to stimulate the cells. Subsequently, cells were incubated with *A. hydrophila* (5×10^6 CFU/mL, 500 μL) for 24 h. The final density of the cell was determined by crystal violet staining.

Fig.S4

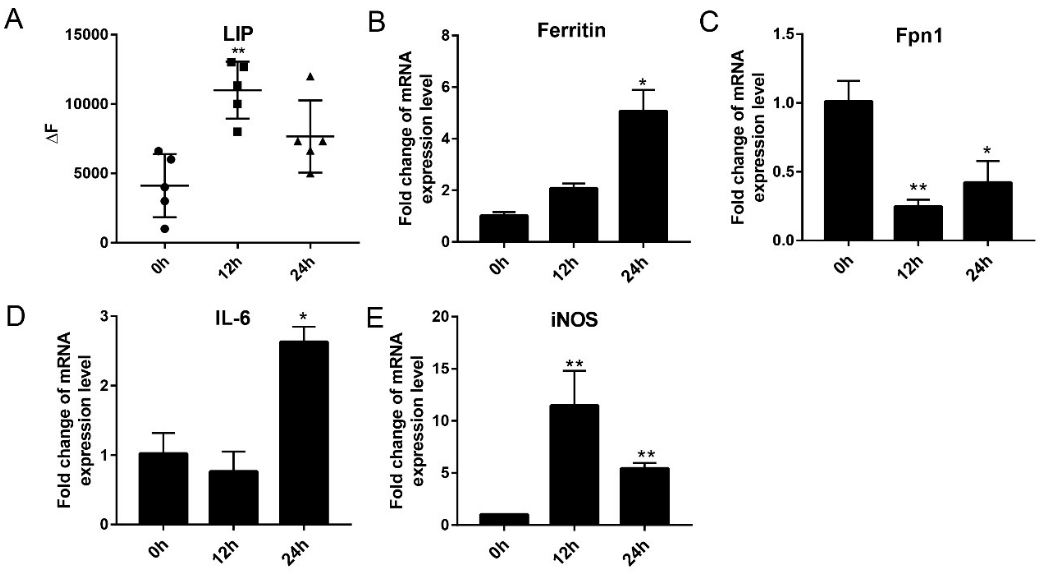

Figure.S4. Effect of bacterial gDNA on iron homeostasis and immune regulation in L8824 cells. (A) The LIP content was detected by fluorometric assay after bacterial DNA stimulation. The cells were harvested for qRT-PCR to quantify the relative expression of Ferritin **(B)**, Fpn **(C)** IL-6 **(D)**, iNOS **(E)**. Data were expressed as mean ± SD (n=4), $^{*}P<0.05$; $^{**}P<0.01$.

Figure.S5

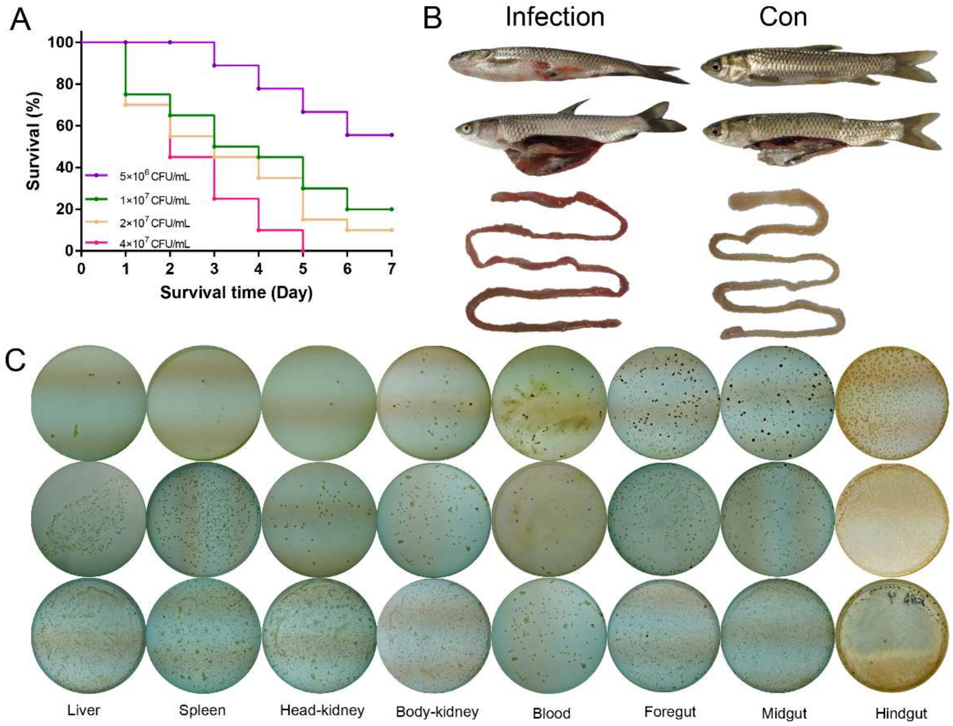

Figure.S5. Grass carp model of intestinal tract infection established by *A. hydrophila* strains. **(A)**The mortality statistics for the grass carp which infected with a linear concentration gradient model of bacteria.**(B)** The symptoms of *A. hydrophila* -infection test in grass carp.**(C)** At 3 days post-infection, the bacterial burden in different tissues of grass carp infected with *A. hydrophila*.
